# Dynamic Solution Space Division-Based Methods for Calculating Reaction Deletion Strategies for Constraint-Based Metabolic Networks for Substance Production: DynCubeProd

**DOI:** 10.3389/fbinf.2021.716112

**Published:** 2021-08-13

**Authors:** Yier Ma, Takeyuki Tamura

**Affiliations:** Bioinformatics Center, Institute for Chemical Research, Kyoto University, Kyoto, Japan

**Keywords:** metabolic network, flux balance analysis, constraint-based model, linear programming, algorithm

## Abstract

Flux balance analysis (FBA) is a crucial method to analyze large-scale constraint-based metabolic networks and computing design strategies for strain production in metabolic engineering. However, as it is often non-straightforward to obtain such design strategies to produce valuable metabolites, many tools have been proposed based on FBA. Among them, GridProd, which divides the solution space into small squares by focusing on the cell growth rate and the target metabolite production rate to efficiently find the reaction deletion strategies, was extended to CubeProd, which divides the solution space into small cubes. However, as GridProd and CubeProd naively divide the solution space into equal sizes, even places where solutions are unlikely to exist are examined. To address this issue, we introduce dynamic solution space division methods based on CubeProd for faster computing by avoiding searching in places where the solutions do not exist. We applied the proposed method DynCubeProd to iJO1366, which is a genome-scale constraint-based model of *Escherichia coli*. Compared with CubeProd, DynCubeProd significantly accelerated the calculation of the reaction deletion strategy for each target metabolite production. In addition, under the anaerobic condition of iJO1366, DynCubeProd could obtain the reaction deletion strategies for almost 40% of the target metabolites that the elementary flux vector-based method, which is one of the most effective methods in existence, could not. The developed software is available on https://github.com/Ma-Yier/DynCubeProd.

## 1 Introduction

Metabolic engineering is a DNA recombination-based technology proposed in 1991 to improve the designated substance production and the cell properties by manipulating and introducing specific biochemical reactions (Bailey, 1991; Stephanopoulos et al., 1998). In many cases, current metabolic engineering technology focuses on the utilization of microorganisms. In metabolic engineering analysis, metabolic pathways in organisms are often represented by metabolic networks, in which nodes represent metabolite molecules and biochemical reactions. Any two metabolites (biochemical reactions) cannot be directly connected, and a metabolite must be connected to at least two biochemical reactions. The biochemical reactions can be irreversible or reversible. Nodes of external metabolites form the input and output nodes of the entire network.

Constraint-based modeling is a mathematical method to identify the best solution within a set of possible choices subject to pre-specified constraints (Maranas and Zomorrodi, 2016). Constraint-based modeling methods, such as linear programming (LP) and mixed integer linear programming, are widely used effective optimization techniques. Flux balance analysis (FBA) is one such widely used constraint-based modeling method with stoichiometric-based modeling of metabolism for the analysis of genome-scale metabolic models (GSMM) (Maranas and Zomorrodi, 2016).

In the constraint-based models of metabolic networks, the cell growth reaction and the target metabolite production reactions are of particular interest. The cell growth reaction has been virtually designed to simulate the efficient conversion of uptake resources into cellular energy and chemical components, which support cell growth in response to selection pressure to construct the system in the most plausible physiological state (Maranas and Zomorrodi, 2016). The target metabolite production reaction produces a chemical of interest. We define growth rate (GR) as cell growth reaction speed and production rate (PR) as the target metabolite production reaction speed.

**FIGURE 1:**
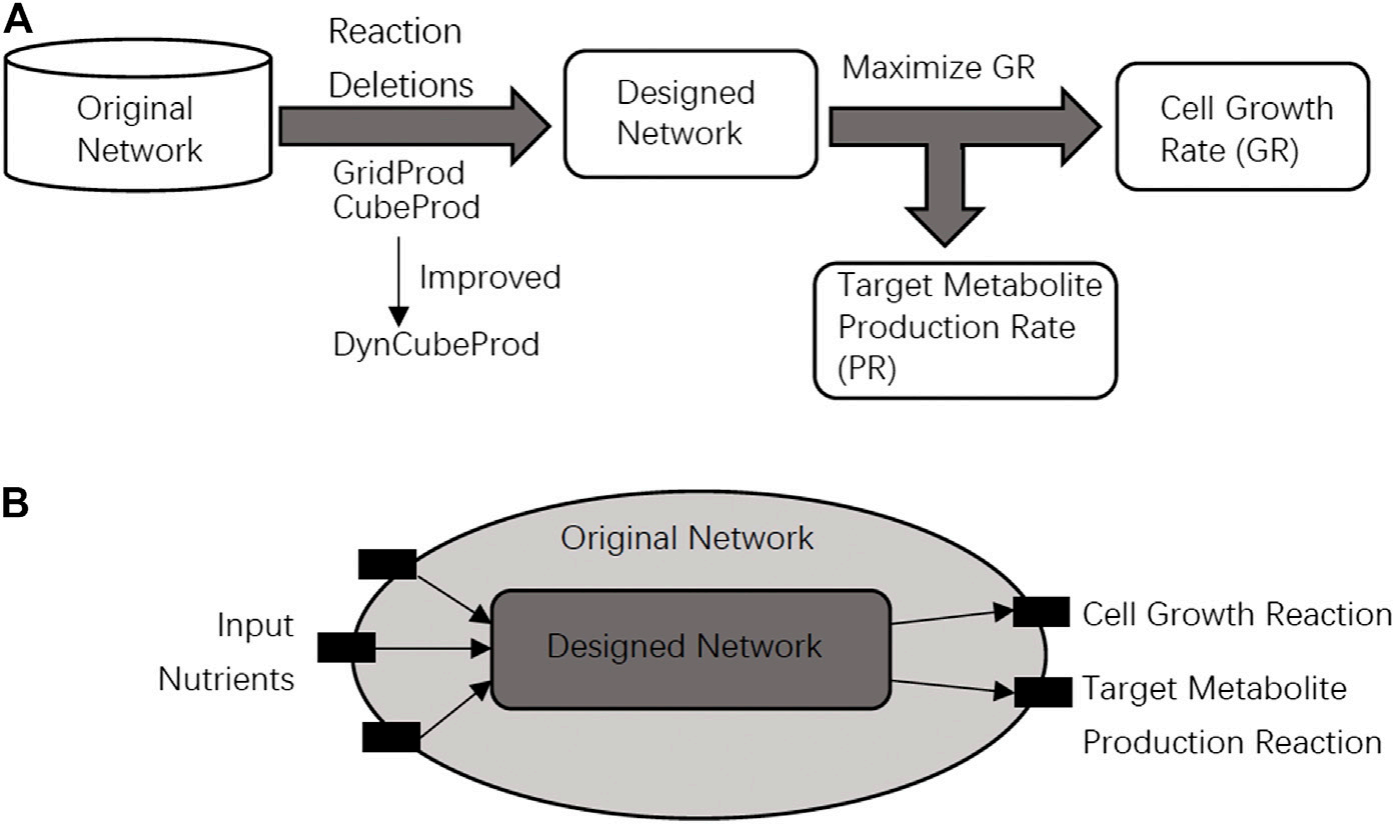
**(A)** In the problem setting of this study, it is required that the original network is converted into the designed network that achieves growth coupling, where cell growth and target metabolite production are simultaneously achieved. **(B)** The designed network should be obtained from the original network through reaction deletions. The black blocks are nutrient uptake reactions, the cell growth reaction, or the target metabolite production reaction. The speed of the cell growth reaction and target metabolite production reaction are represented by GR and PR, respectively. Light gray and dark gray represent the designed network and the original network, respectively.

Growth coupling is a fundamental design principle in metabolic engineering and computational strain design. The purpose of growth coupling is to make the target metabolite a mandatory by-product of the cell growth reaction. We say that growth coupling is achieved if the target metabolite is produced when cell growth is maximized as shown in Figure 1A.

In this study, the core and basic problems are to find a growth coupling method for the target metabolite production through reaction deletions. The method should produce as much target metabolite as possible by modifying the metabolic network when GR is maximized as the objective function. The relationship between the original network and the designed network is shown in Figure 1B. We can delete reactions by setting their speeds as zero in the network modification strategies. Based on the most basic problem above, the following sub-problems are derived. The first is to find knockout strategies for as many different target metabolites as possible. The second is to find knockout strategies for the networks under different input conditions such as aerobic or anaerobic conditions.

The most basic and pioneer algorithm for this purpose is OptKnock, which is a bilevel optimization-based method that identifies knockout strategies that result in the maximum PR when GR is maximized. The inner optimization performs the flux (reaction speed) allocation with regard to the optimization of cellular objectives (e.g., maximization of biomass yield and MOMA) (Burgard et al., 2003). The outer optimization maximizes the bioengineering objective (e.g., chemical production) (Burgard et al., 2003). However, because the computation time of OptKnock is proportional to an exponential function of the network size, in many cases, its computation is not completed within a realistic timeframe for GSMMs (Tamura, 2021b). Therefore, many algorithms have been proposed to speed up the process for the efficient computation of the reaction deletion strategies.

Considering that finding the optimal strategy is NP-hard, it is reasonable to only find out the strategy that meets the expected requirements. For example, the elementary flux vector (EFV)-based method determines reaction deletion strategies in which cell growth forces the production of the target metabolite, and the success ratio of this method was very high under both anaerobic and aerobic conditions for several microbial models (von Kamp and Klamt, 2017). GridProd efficiently computes the design of minimum metabolic networks by using bilevel optimization approach with picking two-dimensional limits and gridding the constraint space (Tamura, 2018). CubeProd divides the entire constraint space into small cubes and gave good results for GSMM with extreme constraints (e.g., anaerobic condition) (Tamura, 2021a). The EFV-based method, GridProd, and CubeProd enable the calculation of reaction deletion strategies for many target metabolites that cannot be calculated using the previously developed methods. However, for *Escherichia coli* under anaerobic conditions, the reaction deletion strategies could not be obtained for many target compounds. In particular, for GridProd and CubeProd, the bottleneck was the computing speed. Therefore, it was expected to extend GridProd or CubeProd to shorten the computation time.

In this study, we developed DynCubeProd that improves the computation speed of CubeProd. DynCubeProd employs a dynamic strategy for the cube sizes to obtain the same results as CubeProd; however its computation speed is much faster. The reaction deletion strategies obtained by DynCubeProd also supplement those of the EFV-based method under certain conditions. Under anaerobic conditions, we obtained the reaction deletion strategies for close to 40% of the target metabolites for which the EFV-based method could not determine strategies.

## 2 Materials and Methods

### 2.1 Problem Definition

The general formalization of constraint-based modeling is as follows (Maranas and Zomorrodi, 2016):

**minimize (or maximize):**

f(x)

**subject to:**

h(x)=0

g(x)≤0

x∈S

x is an n -dimensional variable. f(x) is the objective function to minimize or maximize. S is the set from which the variable vector x ranges. h(x) and g(x) are the constraints that must be satisfied as equalities or one-side inequalities, respectively.

The general form of the FBA is as follow:

**maximize**

f(x)

**subject to:**

Sx=0

LB≤x≤UB

$x\in R^{n}$ is an n -dimensional variable. f(x) is the objective function, which in many cases is GR. $S\in R^{m\times n}$ is the stoichiometric matrix corresponding to m metabolites and n reactions in the constraint-based models. LB and UB impose the lower and upper bounds of each x∈x . For example, a flux for irreversible reactions $x_{i}$ is constrained as $x_{i}\geq 0$ .

Our goal was to find reaction deletion strategies for growth coupling of target metabolite production. Let $K=\left\{v_{j}|v_{j}\in V\right\}$ be a set of reactions to be knocked out, where V is a set of n reactions. Then, the definition of the main problem of this study arises.

**Given**

$\;S,LB,UB,v_{growth},v_{target},x_{growth}^{min},x_{target}^{threshold}$

**Find**

  K

  **such That**

${\;x}_{growth}\geq x_{growth}^{min}$ and $x_{target}\geq x_{target}^{threshold}$

  **maximize**

f(x) (= $x_{growth}$ )   **subject to:**

Sx=0

$\left\{\begin{array}{c} x=0\;if\;,\;x\in K\;\\ LB\leq x\leq UB,\;\;otherwise.\; \end{array}\right.$

When $x_{growth}\geq x_{growth}^{min}$ and $x_{target}\geq x_{target}^{threshold}$ is satisfied, we consider K achieves growth coupling, where $GR=x_{growth}$ for $v_{growth}\in V$ and $PR=x_{target}$ for $v_{target}\in V$ hold.

### 2.2 Example for Problem Definition

**FIGURE 2:**
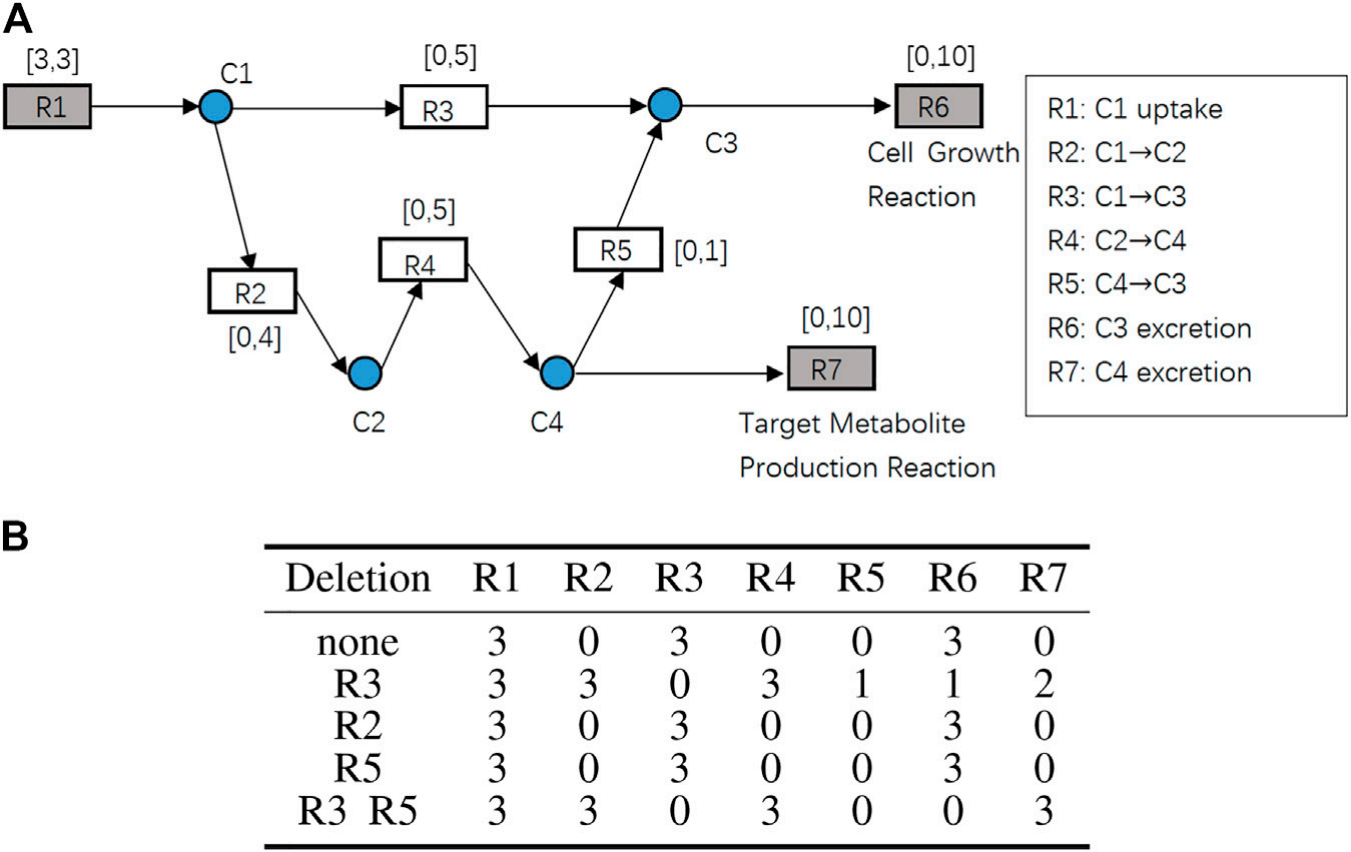
**(A)** A toy example of the constraint-based models. Rectangular nodes R1 to R7 are reactions, and the attached intervals represent lower and upper bounds of their reaction speeds. R1 is the nutrient uptake reaction. R6 is the cell growth reaction. R7 is the target metabolite production reaction. Circular nodes C1 to C4 are internal metabolites. **(B)** Reaction deletion strategies and the resulting flux (reaction speed) distributions.

A toy example of the constraint-based model with 11 nodes is shown in Figure 2A to illustrate the problem definition explained above. The rectangular nodes {R1,R2,…,R7} are chemical reactions. R7 is the target metabolite production reaction and R6 is the cell growth reaction. The substrates and products of the reactions are shown on the right side of Figure 2A. The gray rectangular nodes are external reactions which play roles of input and output of the entire network and the white rectangular nodes are internal reactions, each of which connect at least two metabolite nodes with different directions. The intervals next to the rectangular nodes are the lower and upper bounds of reaction speeds. The circular nodes are internal metabolites that connect rectangular nodes.

Suppose that $v_{growth}^{min}=v_{target}^{threshold}=1$ is given. When a reaction deletion strategy is given and $GR=x_{6}$ is maximized, if GR≥1 and $PR=x_{7}\geq 1$ hold, then we consider that growth coupling is achieved. Because this example is very simple, such a reaction deletion strategy can be easily determined through brute force enumeration. Deleting R3 is the optimal solution for this toy example.

Figure 2B shows the results of each knockout strategy applied to this toy example. Because deleting R4 is practically equivalent to deleting R2, deleting R4 is omitted in the table. When none of the reactions are deleted, that is, K=ϕ , GR = 3 will be obtained but PR will be 0. When K={R2} or K={R5} , the same result will be obtained. According to the definition of the problem above, such knockout strategies are not acceptable. When K={R3,R5} , PR = 3 is obtained, but GR will be 0. When K={R3} , both GR=1≥1 and PR=2≥1 are obtained. Therefore, K={R3} is a feasible solution because it achieves growth coupling. However, such a brute force method cannot be applied to GSSMs owing to combinatorial explosion.

## 3 Results

We developed an algorithm DynCubeProd for calculating reaction deletion strategies that achieve growth coupling of designated target metabolite production in constraint-based models of metabolic networks.

Because DynCubeProd is a method obtained by improving CubeProd (Tamura, 2021a), in this section, we provide an overview of CubeProd and then explain the difference between DynCubeProd and CubeProd. The algorithm behavior of DynCubeProd is also illustrated using examples. The relationship between DynCubeProd and other methods is discussed in Section 4.

### 3.1 DynCubeProd

Idea: CubeProd considers the three-dimensional solution space whose axes represent GR, PR and sum of absolute values of fluxes (SF). Let TMGR, TMPR, and TMSF be the theoretical maximum values of the above, respectively. Then the whole constraint space is a rectangle formed by [0, TMGR], [0, TMPR], and [0,TMSF]^1^. According to the designated value of P , each of [0, TMGR], [0, TMPR], and [0,TMSF] are divided into P pieces. Therefore, finally, CubeProd considers $P^{3}$ constraint sub-spaces.

The value of P closely affects the trade-off between the ease of finding a solution and the computation time. The larger the value of P, the easier it is to find a solution, but the slower the computation time. Therefore, if CubeProd uses a certain value of P and cannot find a solution, a larger value of P should be applied, but the computation time will be more. However, it may be the case that some of the small solution spaces generated by a larger P are already proved by a smaller P to contain no solutions.

DynCubeProd starts with *p* = 1 and doubles P if no solution is found. When applying a larger P, it refers to the result of applying the smaller P and avoids searching for places where there is no solution.

Because the intervals on each of the three axes are equally subdivided into P sub-intervals, the entire constraint space is divided into $P^{3}$ sub-spaces, and

$$\frac{\left(i-1\right)\times TMGR}{P}\leq x_{growth}\leq \frac{i\times TMGR}{P},$$

$$\frac{\left(j-1\right)\times TMPR}{P}\leq x_{target}\leq \frac{j\times TMPR}{P},$$

$$\frac{\left(k-1\right)\times TMSF}{P}\leq \sum |x|\leq \frac{k\times TMSF}{P}$$

are added as constraints and the sum of the absolute values of fluxes is minimized for every 1≤i,j,k≤P , where i,j,k are integers.

In each of those $P^{3}$ sub-spaces, 1) LP is employed with the above three constraints, 2) if the LP is feasible, reactions whose flux is less than $10^{-5}$ are collected as K , 3)the minimum value of PR is calculated with deletions of K under the condition that GR is maximized without the above three constraints, and 4) if GR and PR exceed the minimum required values, the output K is considered as the solution.

The number of sub-spaces to be computed is $P^{3}$ and it increases dramatically as P increases, which will lead to a power-of-three increase in computation time. However, the larger the value of P , the smaller is the range of the subspace and the easier it is to approach the point of the optimal solution or local optimal solution.

A dynamic strategy is adopted by DynCubeProd to save time. Starting with i=1 for $P=2^{i}$ , DynCubeProd increases i one by one, and stops once an acceptable knockout strategy is obtained. Suppose that Q is a sub-space corresponding to P=m and $\left\{Q_{1},\ldots ,Q_{k}\right\}$ are sub-spaces of Q for P=n with m<n . If the candidate knockout strategy computed from Q is not acceptable when P=m , all the sub-spaces $\left\{Q_{1},\ldots ,Q_{k}\right\}$ will be skipped during the calculation when P=n .

Example:
Table 1A represents the whole solution space by DynCubeProd with P=1 of the toy network of Figure 2A whereas Table 1B represents all sub-spaces for P=2 . For each sub-space, LPs are performed twice; first, to calculate the possible candidate knockout strategy and second, to verify that the candidate is acceptable. The results of two LPs are represented for each (sub)space in Tables 1A,B.

**TABLE 1:** DynCubeProd applies CubeProd beginning with *p* = 1 and increments one by one and quits when a desired reaction deletion strategy is obtained. **(A)** and **(B)** represent the results of DynCubeProd applied to the network of Figure 2A with *p* = 1 and *p* = 2, respectively.

	(A) CubeProd (*p* = 1)									
	PR	GR	SF	R1	R2	R3	R4	R5	R6	R7
	[0,3]	[0,3]	[0,26]	3	0	3	0	0	3	0
				3	0	3	0	0	3	0
	**(B) CubeProd (*p* = 2)**									
Sub-space ID	PR	GR	SF	R1	R2	R3	R4	R5	R6	R7
1	[0,1.5]	[0,1.5]	[0,13]	3	1.5	1.5	1.5	0	1.5	1.5
				3	0	3	0	0	3	0
2	[0,1.5]	[0,1.5]	[13,26]	null	null	null	null	null	null	null
3	[0,1.5]	[1.5,3]	[0,13]	3	0	3	0	0	3	0
				3	0	3	0	0	3	0
4	[0,1.5]	[1.5,3]	[13,26]	null	null	null	null	null	null	null
5	[1.5,3]	[0,1.5]	[0,13]	3	1.5	1.5	1.5	0	1.5	1.5
				3	0	3	0	0	3	0
6	[1.5,3]	[0,1.5]	[13,26]	null	null	null	null	null	null	null
7	[1.5,3]	[1.5,3]	[0,13]	3	1.5	1.5	1.5	0	1.5	1.5
				3	0	3	0	0	3	0
8	[1.5,3]	[1.5,3]	[13,26]	null	null	null	null	null	null	null

As shown in Table 1A, when P=1 , the number of sub-spaces is one; this implies that the sub-space is equivalent to the entire original constraint space. As TMGR, TMPR, and TMSF are 3, 3, 13, respectively, Three constraints 0≤GR≤3 , 0≤PR≤3 and 0≤SF≤2⋅13 are added and the sum of absolute values of fluxes is minimized in the first LP. Then R1 = R3 = R6 = 3 and R2 = R4 = R5 = R7 = 0 are obtained. Therefore, K={R2,R4,R5} is obtained as a reaction deletion strategy candidate because the target metabolite production reaction R7 is not allowed to be deleted. K is validated by the second LP, however, GR = 3 and PR = 0 are obtained. Therefore, the deletion strategy K={R2,R4,R5} is not acceptable because PR does satisfy the minimum required value $x_{target}^{threshold}=1$ .

Therefore, DynCubeProd with *p* = 2 is applied. The results are summarized in Table 1B. Because the number of axes in DynCubeProd is three, eight sub-spaces are created when P=2 . For the sub-space IDs 2, 4, 6, and 8 in Table 1B, there is no feasible solution in the first LP. Therefore, the computing in P=4 will be skipped for all sub-spaces of the sub-spaces whose IDs are 2, 4, 6, and 8 in P=2 . This process of skipping computing the sub-spaces that do not contain solutions makes DynCubeProd computation faster than CubeProd. DynCubeProd will end the computing and return results either when P reaches the designated maximum value or when an acceptable solution is obtained.

Lemma 1:
*For a positive integer* k *, the solution obtained by DynCubeProd for* P=2k *is also a solution for* P=k *, and the constraint sub-spaces skipped by DynCubeProd do not include a solution.*

Proof:
Define S as the constraint space of an LP problem. Let $S_{1}\cup S_{2}\cup \cdots \cup S_{n}=S$ hold. Suppose there exists a solution x in the sub-space $S_{k},\;x\in S_{k}$ . Then, x must be in the space of S,x∈S , because $S_{k}\subset S$ . If there is no solution x in S , that is, x∉S , then such x must not exist in any sub-space of S .

### 3.2 Pseudo Code of DynCubeProd

The pseudo code of DynCubeProd is as follow:

Algorithm 1:
DynCubeProd

[K,PR,GR]= DynCubeProd ( S,LB,UB,GUR,OUR,NGAM ,

$v_{growth},v_{target},x_{growth}^{min},x_{target}^{threshold},n_{max}$ )

$TMGR=max\;x_{growth}$

$\;s.t.\;\sum S_{i,j}x_{j}=0$

$\;\;LB_{j}\leq x_{j}\leq UB_{j}$

$\;\;x_{glc\text{\_}uptake}\geq -GUR$

$\;\;x_{o2\text{\_}uptake}\geq -OUR$

$\;\;x_{atp\text{\_}main}\geq NGAM$

$TMPR=max\;x_{target}$

$\;s.t.\;\sum S_{i,j}x_{j}=0$

$\;\;LB_{j}\leq x_{j}\leq UB_{j}$

$\;\;x_{glc\text{\_}uptake}\geq -GUR$

$\;\;x_{o2\text{\_}uptake}\geq -OUR$

$\;\;x_{atp\text{\_}main}\geq NGAM$

$\;\;x_{growth}\geq x_{growth}^{min}$

$\;\;TMSF=\sum |x_{j}|$

c(1,1,1)=1

n=1

**while** true **do**

$\;P=2^{n}$

d=zeros(P,P,P)

 **for** i=1toP/2 **do**
  **for** j=1toP/2 **do**
   **for** k=1toP/2 **do**

d(2i,2j,2k)=c(i,j,k)

d(2i,2j,2k−1)=c(i,j,k)

d(2i,2j−1,2k)=c(i,j,k)

d(2i,2j−1,2k−1)=c(i,j,k)

d(2i−1,2j,2k)=c(i,j,k)

d(2i−1,2j,2k−1)=c(i,j,k)

d(2i−1,2j−1,2k)=c(i,j,k)

d(2i−1,2j−1,2k−1)=c(i,j,k)

     **end for**
   **end for**
**end for**
**for** i=1toP **do**

biomassLB=TMGR×P×(i−1)

biomassUB=TMGR×P×i >
**for** j=1toP **do**

targetLB=TMPR×P×(j−1)

targetUB=TMPR×P×j

**for** k=1toP **do**

sumfluxLB=2×TMSF×P×(k−1)

sunfluxUB=2×TMSF×P×k

**if** d(i,j,k)=0 t**hen**

continue

**else**

$min\;\sum |x_{b}|$

$\;s.t.\;\sum S_{a,b}x_{b}=0$

$\;\;LB_{b}\leq x_{b}\leq UB_{b}$

$\;\;x_{glc\text{\_}uptake}\geq -GUR$

$\;\;x_{o2\text{\_}uptake}\geq -OUR$

$\;\;x_{atp\text{\_}main}\geq NGAM$

$\;\;biomassLU\leq x_{growth}\leq biomassUB$

$\;\;targetLB\leq x_{target}\leq targetUB$

$\;\;sumfluxLB\leq \sum |x_{b}|\leq sumfluxUB$

$\;\;K=\left\{v_{b}|x_{b}<10^{-5}\right\}$

**If** thefirstLPisinfeasible **then**
       
K=ϕ

       
PR(i,j,k)=0

       
c(i,j,k)=0

**else**

$max\;x_{growth}$

$\;s.t.\;\sum S_{a,b}x_{b}=0$

$\;\;LB_{b}\leq v_{b}\leq UB_{b}$ if $v_{b}\notin K$

$\;\;x_{b}=0\;if\;v_{b}\notin K$

$\;\;x_{glc\text{\_}uptake}\geq -GUR$

$\;\;x_{o2\text{\_}uptake}\geq -OUR$

$\;\;x_{atp\text{\_}main}\geq NGAM$

c(i,j,k)=1

**if** $x_{target}\geq x_{target}^{threshold}$ and $x_{growth}\geq x_{growth}^{min}$ **then**
        **return** K , $x_{target}$ , $x_{growth}$

        **end if**
       **end if**
     **end if**
    **end for**
  **end for**
 **end for**
**if** $n=max_{n}$ t**hen**
   
break

**end if**

n=n+1

**end while**

### 3.3 Computational Experiments

The dataset used in the computational experiments was iJO1366, which is a GSMM of *Escherichia coli* K-12 MG1655 from the BiGG database with 1805 metabolites and 2583 reactions (Orth et al., 2011; King et al., 2016). All procedures of DynCubeProd were implemented based on Gurobi, COBRA Toolbox and MATLAB on a Windows machine with Intel(R) Core(TM) i5-8500 CPU 3.00 GHz 6-core processor and 32.0 GB RAM.

If the target metabolite is not connected to an external reaction, then, an auxiliary external reaction is added, and the growth coupling is evaluated by GR and the outgoing flux from the additional external reaction, which is also called PR.

**FIGURE 3:**
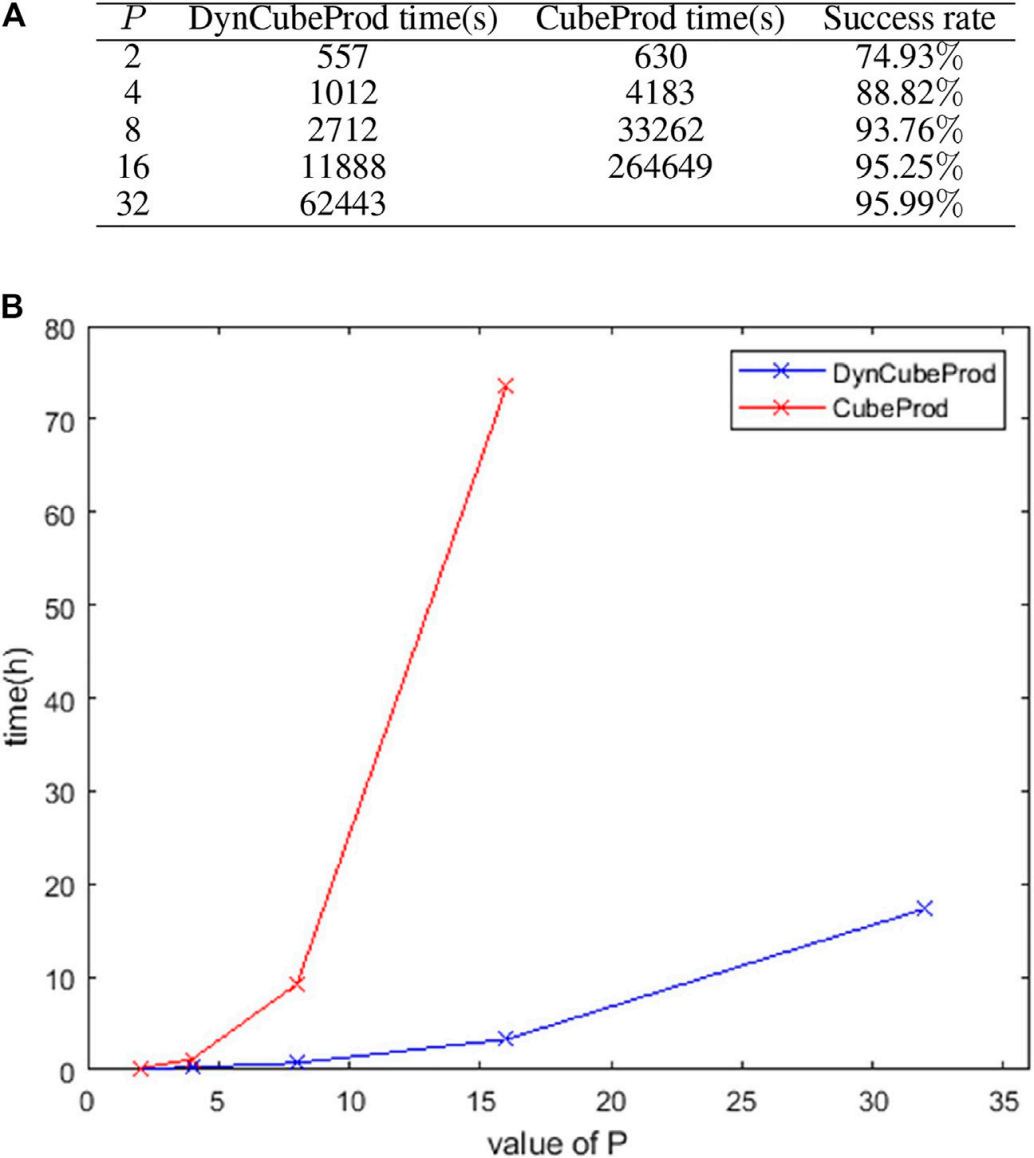
**(A)** Computation time and success ratio when DynCubeProd and CubeProd were applied to iJO1366 under aerobic conditions for different values of P . **(B)** Visual comparison of the computation time of DynCubeProd and CubeProd of (A).

Figure 3A shows the computing time of DynCubeProd and CubeProd when applied to iJO1366 under aerobic conditions at different values of P. It also shows the ratio of the number of success cases to the number of target metabolites. For P≥16 , the reaction deletion strategies were obtained for more than 95% of the target metabolites. It should be noted that the success ratio of DynCubeProd and CubeProd is always the same for the same P. The computing time for DynCubeProd with *p* = 32 was only approximately quarter of the computing time of CubeProd with *p* = 16. Figure 3B visually compares the computing time increase by P between DynCubeProd and CubeProd.

Furthermore, under anaerobic conditions of iJO1366, DynCubeProd succeeded in computing the reaction deletion strategies for 76 of the 211 target metabolites for which the EFV-based method (von Kamp and Klamt, 2017), which is one of the best methods, could not.

## 4 Discussion

To date, numerous algorithms have been proposed using constraint-based models to compute reaction deletion strategies for growth coupling in GSMM. In particular, the EFV-based method is one of the most efficient methods to determine reaction deletion strategies that achieve growth coupling of target metabolites. The success ratios of the EFV-based method for iJO1366 under aerobic and anaerobic conditions were 99.4 and 77.4%, respectively (von Kamp and Klamt, 2017). Because the success ratio of DynCubeProd for iJO1366 under aerobic conditions was 95.99% for *p* = 32, the EFV-based method is more efficient under aerobic conditions and there is almost no room for improvement.

However, for iJO1366 under anaerobic conditions, the success ratio of the EFV-based method is 77.4%. An effective method for target metabolites for which reaction deletion strategies could not be calculated by the EFV-based method must be developed. Because DynCubeProd succeeded in determining the reaction deletion strategies for 76 of such 211 target metabolites as described in Section 3, we can conclude that DynCubeProd can play a complementary role to the EFV-based method in the calculation of reaction deletion strategies for growth coupling.

The success ratio of DynCubeProd and CubeProd is always the same if P is the same. However, since DynCubeProd is faster than CubeProd, DynCubeProd can reach a larger P in realistic computation time. Therefore, if the allowable computation time is the same, DynCubeProd is more capable of computing reaction deletion strategies than CubeProd. The speed-up of the computation is achieved by not examining the constraint space where no solution exists, and by stopping the algorithm as soon as a solution satisfying the requirements is obtained. Additionally, CubeProd is a method developed by (Tamura, 2021a); however in this study, the definition of TMSF is slightly modified to be the absolute sum of reaction rates at PR maximization.

If the computation is conducted for very large $P=2^{i}$ , the computation time of DynCubeProd becomes too long. Therefore, values up to $P=2^{i}=32$ were used in the computational experiments in this study. Even if the computation is conducted for all P= 2, 4, 8, 16, and 32, the total number of cubes can be regarded as not too large to conduct several LPs for each cube and as an ignorable constant when estimating the computational complexity of DynCubeProd. It is known that an LP can be solved in a time proportional to the polynomial function of the number of variables (Arora and Barak, 2009). Because DynCubeProd solves several LPs for the entire problem and for each cube, the computational complexity of the algorithm is proportional to the polynomial function of the problem size because P can be regarded as a constant.

### 4.1 Comparison With GridProd and CubeProd

DynCubeProd was developed as an extension of CubeProd that was developed as an extension of GridProd. Therefore, a problem example that can be solved with CubeProd, but not with GridProd, may help understand for DynCubeProd. Such an example is illustrated below.

GridProd picks two-dimensional limits and grids the constraint space to solve the problem by using bilevel optimization approach (Tamura, 2018). Table 2A shows example results of GridProd applied on the toy network. GR and PR are selected as the two-dimensional limits of GirdPord, the gridding of the GridProd will be performed on the axes of these two constraints, and the size of the gridding depends on the designated value of P . In Table 2A, the second and third rows show results of the bilevel optimization approach of a grid with *p* = 4, that is, GR ∈[1.5,2.25] and PR ∈[0.75,1.5] . The grid becomes a point when P is infinite. The fourth and fifth rows represent the results in the point of optimal solution when P is infinite. The fifth row shows that the acceptable solution cannot be obtained even when P is under infinity.

**TABLE 2:** **(A)** GridProd cannot find reaction deletion strategies that achieve growth coupling for the toy example of Figure 2A even when P is infinity. **(B)** However, CubeProd can find reaction deletion strategies for the same problem

(A)									
PR	GR		R1	R2	R3	R4	R5	R6	R7
[1.5,2.25]	[0.75,1.5]	1st LP	3	1.5	1.5	1.5	0	1.5	1.5
		2nd LP	3	0	3	0	0	3	0
[2,2]	[1,1]	1st LP	3	2	1	2	0	1	2
		2nd LP	3	0	3	0	0	3	0
**(B)**									
**PR**	**GR**	**SF**	**R1**	**R2**	**R3**	**R4**	**R5**	**R6**	**R7**
[1.5,2.25]	[0.75,1.5]	[12,14]	3	2.25	0.75	2.25	0.75	1.5	1.5
			3	0	3	0	0	3	0
[2,2]	[1,1]	[13,13]	3	3	0	3	1	1	2
			3	3	0	3	1	1	2

CubeProd, which is an extension of GridProd, divides the entire constraint space into small cubes (Tamura, 2021a). Table 2B depicts a sample result of applying CubeProd to the toy network. The second to fifth rows show the results of bilevel optimization for cubes containing the optimal solution for different values of P. Since GR = 3 and PR = 0 are obtained, CubeProd cannot obtain the reaction deletion strategy for growth coupling for PR ∈ [1.5,2.25], GR ∈ [0.75,1.5], and SF ∈ [12,14]. However, since GR = 1 and PR = 2 are obtained, CubeProd can obtain the reaction deletion strategy for growth coupling for PR ∈ [2,2], GR ∈ [1,1], and SF ∈ [13,13]. As shown in this example, the larger the value of P and smaller the cube sizes, the easier it is to find a solution.

### 4.2 Other Methods

For the problem of calculating reaction-deletion strategies for growth coupling of the constraint-based models, there are various methods other than those introduced so far. OptGene is a computational method that uses bio-inspired algorithms to optimize gene deletion sets (Patil et al., 2005). Genetic Design through Local Search (GDLS) was developed to use global optimal search to find genetic design, and compared with heuristic search based on evolutionary algorithm and simulated annealing, GDLS performs well (Lun et al., 2009). EMILiO uses iterative linear programs (Yang et al., 2011). FastPros evaluates the potential of specific reaction knockout to produce specific metabolites by shadow pricing the constraints in FBA, thereby generating a screening score to obtain candidate knockout sets (Ohno et al., 2014). IdealKnock can effectively evaluate the production potential of different biochemical products in the system, just by knocking out some pathways and combining with the OptKnock or OptGene framework (Gu et al., 2016). Parsimonious enzyme usage FBA (pFBA) not only used the metabolic network, but also used proteomics and transcriptomics data to confirm that almost all path dosages predicted by the FBA optimization method were consistent (Lewis et al., 2010). IdealKnock utilizes the concept of ideal-type flux distribution (Gu et al., 2016). PSOMCS also uses the perspective of EFM, combined with particle swarm optimization algorithms, to obtain an optimal design that meets multiple goals (Nair et al., 2017).

For the performance evaluation of DynCubeProd, it is reasonable to compare it with GridProd and CubeProd for the evaluation of computation time reduction and algorithm behavior comparison, and it is reasonable to compare it with the EFV-based method, a method with the best success rate among existing methods, for the evaluation of success rate. Furthermore, neither OptKnock nor GDLS, one of the most standard and popular methods, could determine reaction deletion strategies for any target metabolite within 1 hour. Therefore, although there are many other existing methods as mentioned above, it is reasonable to narrow down the comparison targets of DynCubeProd to GridProd, CubeProd, and EFV methods.

### 4.3 Conclusion

DynCubeProd is an improved version of CubeProd, which is an existing algorithm based on solution space decomposition. The improvements in DynCubeProd are as follows. 1) While CubeProd divides the solution space based on pre-specified parameters, DynCubeProd gradually divides the solution space into smaller and smaller pieces. 2) CubeProd mechanically explores even the subspace where no solution is expected to exist, while DynCubeProd stops dividing the solution space when no solution exists. 3) While CubeProd searches the entire solution space, DynCubeProd stops as soon as it finds a solution that meets the conditions. The results of computer experiments using iJO1366 confirmed that DynCubeProd reduces the computation time more than 10 times than CubeProd. The reduction in computation time enabled finer solution space partitioning, and reaction deletion strategies could be calculated for about 40% of the target metabolites for which reaction deletion strategies could not be obtained by the EFV-based method. In this study, we developed DynCubeProd, by improving the computation speed of CubeProd, which enabled us to calculate reaction deletion strategies in anaerobic conditions for many target compounds that could not be calculated before.

## Data Availability

The developed software is available on https://github.com/Ma-Yier/DynCubeProd.

## Footnotes

1 Because it is difficult to determine TMSF in polynomial time, we approximate TMSF by SF when PR is maximized, and [0,2⋅TMSF] was used instead of [0,TMSF] in the computational experiments and examples.
